# Implementation mechanisms used in national efforts to improve community services to keep individuals with mental illness out of local jails

**DOI:** 10.1186/s43058-025-00835-5

**Published:** 2025-12-13

**Authors:** Niloofar Ramezani, Faye S. Taxman, Benjamin J. Mackey, Jill Viglione, Jennifer E. Johnson

**Affiliations:** 1 Department of Biostatistics, Virginia Commonwealth University, 830 East Main Street, Box 980032, Richmond, VA USA; 2 Center for Advancing Correctional Excellence, Schar School of Policy & Government, George Mason University, 4400 University Drive, Fairfax, VA USA; 3 Department of Criminal Justice, University of Central Florida, 12494 University Blvd., Orlando, FL USA; 4 Charles Stewart Mott Department of Public Health, Michigan State University, 200 East 1 st Street, Flint, MI USA

**Keywords:** Implementation Mechanisms, Relationship-Building, Performance Monitoring, Interagency Coordination, Justice-Health Reforms

## Abstract

**Background:**

Little is known about effective implementation processes by which counties can improve treatment services to keep people with mental illness and substance use disorders out of local jails. This study examines hypothesized implementation mechanisms (relationship building, performance monitoring, interagency coordination, capacity building, and infrastructure programming) as predictors of outcomes (improved community services) and as mediators of the effects of a national implementation intervention (Stepping Up [SU]), on community services.

**Methods:**

A survey was conducted of mental health, substance use, jail, and probation administrators in 519 U.S. counties, of which 328 counties participated in a national jail reform effort (SU). Survey data were combined with descriptive data from the U.S. Census Bureau. Predictors included hypothesized implementation mechanisms (performance monitoring, interagency coordination teams, creating integrated systems of care, capacity building, relationship building, and quality programming). Covariates included county sociodemographic characteristics (e.g., size of county, size of jail, etc.) and general county service characteristics (e.g., primary care physicians per capita, Medicaid expansion). Implementation outcomes included number of evidence-based practices (EBPs) and evidence-based mental health treatments (MH-EBTs) for individuals with mental illness involved with justice systems. Multilevel regression analyses examined cross-sectional: (1) effects of Stepping Up on outcomes; (2) effects of implementation mechanisms on implementation outcomes; and (3) implementation mechanisms as mediators of the effects of Stepping up on implementation outcomes.

Findings.

SU was found to significantly predict the number of EBPs and MH-EBTs controlling for various demographic characteristics of the counties. When implementation mechanisms were added to these models, SU is no longer statistically significant. Instead, two implementation mechanisms (performance monitoring and interagency coordination) and Medicaid funding significantly predicted the availability of both EBP and/or MH-EBT. Other factors that predicted MH-EBTs include relationship building size of the county, rate of primary care physicians, rate of MH providers in the county, and jail population size. Mediation models found that SU significantly predicted these evidence-based outcomes through implementation mechanisms except interagency coordination.

**Conclusions:**

Little is known about the implementation mechanisms to decarcerate and build programming for mental health services in a county. SU is an important attribute to facilitate reform both directly and indirectly through implementation mechanisms. Counties can benefit from use of relationship building activities to advance policy and service reform efforts, identifying performance metrics of their system, and having infrastructure available to improve the availability of EBPs. Overall, policy changes are possible, but an emphasis should be on strategies that increase the availability of EBPs and MH-EBTs.

**Supplementary Information:**

The online version contains supplementary material available at 10.1186/s43058-025-00835-5.

Contributions to the literature

- Criminal justice–health reforms can employ a variety of implementation strategies commonly used in public health practice, including relationship-building, performance monitoring, interagency coordination, capacity building, and infrastructure programming.
- Reforms that use performance monitoring and interagency coordination, and rely on relationship-building strategies, have the greatest impact on improving service availability, including community mental health treatment services.
- This study of implementation mechanisms illustrates the importance of how agencies pursue change in community health services.

## Introduction

With more than 8 million admissions to local jails in the U.S. each year, people with mental health disorders tend to be overrepresented due to a variety of factors including the dearth of available mental health (MH) treatment services and facilities in the community, lack of diversion to treatment initiatives, and state laws that emphasize incarceration [1–3]. People with MH conditions experience jail incarceration more often, spend more time in jail, and are more likely to return to jail than individuals without mental illness [4]. Reducing the use of jail for people with mental health problems is a complex policy issue, often requiring interagency efforts to affect who is detained in jail; available resources for mental health treatment programs and services in the jail and community; support for jail diversion from law enforcement, judicial actors, and community groups, among others; and coordination between in-jail and community-based treatment services. Each area is a challenge, but collectively decarceration policies and practices require a commitment to identify the individuals with MH issues and divert them from jail using community-based treatment services.

Johnson and colleagues [5] documented 18 recommended evidence-based practices and 42 MH treatments to manage people with mental illness by diverting them from jail and/or provide treatment to improve the individuals’ functioning (see Supplemental Table 2). Each is offered in only 22–43% of U. S. counties [5]. The low uptake of these practices and treatments attests to the difficulties in improving service delivery as a tool to decarcerate local jails.

This paper examines (1) the impact of one national initiative (Stepping Up [SU]) to reform how jails are used on outcomes related to type of programs and services offered; (2) the influence of implementation mechanisms on outcomes; and (3) the role of implementation mechanisms as mediators of Stepping Up on outcomes. Stepping Up was a 2015–2025 reform strategy offered by the Justice Policy Center of the Council of State Government, National Association of Counties, and the American Psychiatric Association Foundation. Stepping Up offered a formula for decarceration to include: (1) organize a policy team of diverse agencies; (2) adopt a mental health screening tool and review the data; (3) assess capacity to provide services; (4) track data on four key measures to asses impact of their efforts over time (e.g., number of people with mental illnesses who are booked into jail, average length of stay, percentage of people connected to community-based treatment after release from jail, and rate of return to jail); (5) develop and implement a plan to improve service delivery; and (6) prioritize funding for programs and services to implement the plan based on performance measures. The national technical assistance agencies worked with counties on this planning and implementation strategy. Over 583 counties in the U.S. joined the Stepping Up Initiative by signing a resolution with commitment to Stepping Up decarceration goals [6]. The goal is to reduce the size of local jail populations by building the capacity of county service systems to provide evidence-based mental health and substance use care. To assist counties, Stepping Up technical assistance organizations provide online technical assistance including webinars, resource materials, toolkits, mentoring sites, and specialized assistance for performance metric development. The focus is primarily on interagency teams, performance monitoring, and MH treatment services. Stepping Up is open to any county that passes a resolution to participate in the initiative. The desired implementation outcomes —namely, the number of available EBPs and the number of behavioral health treatments available to justice-involved individuals—are the primary outcomes examined in this study.

**Fig. 1:**
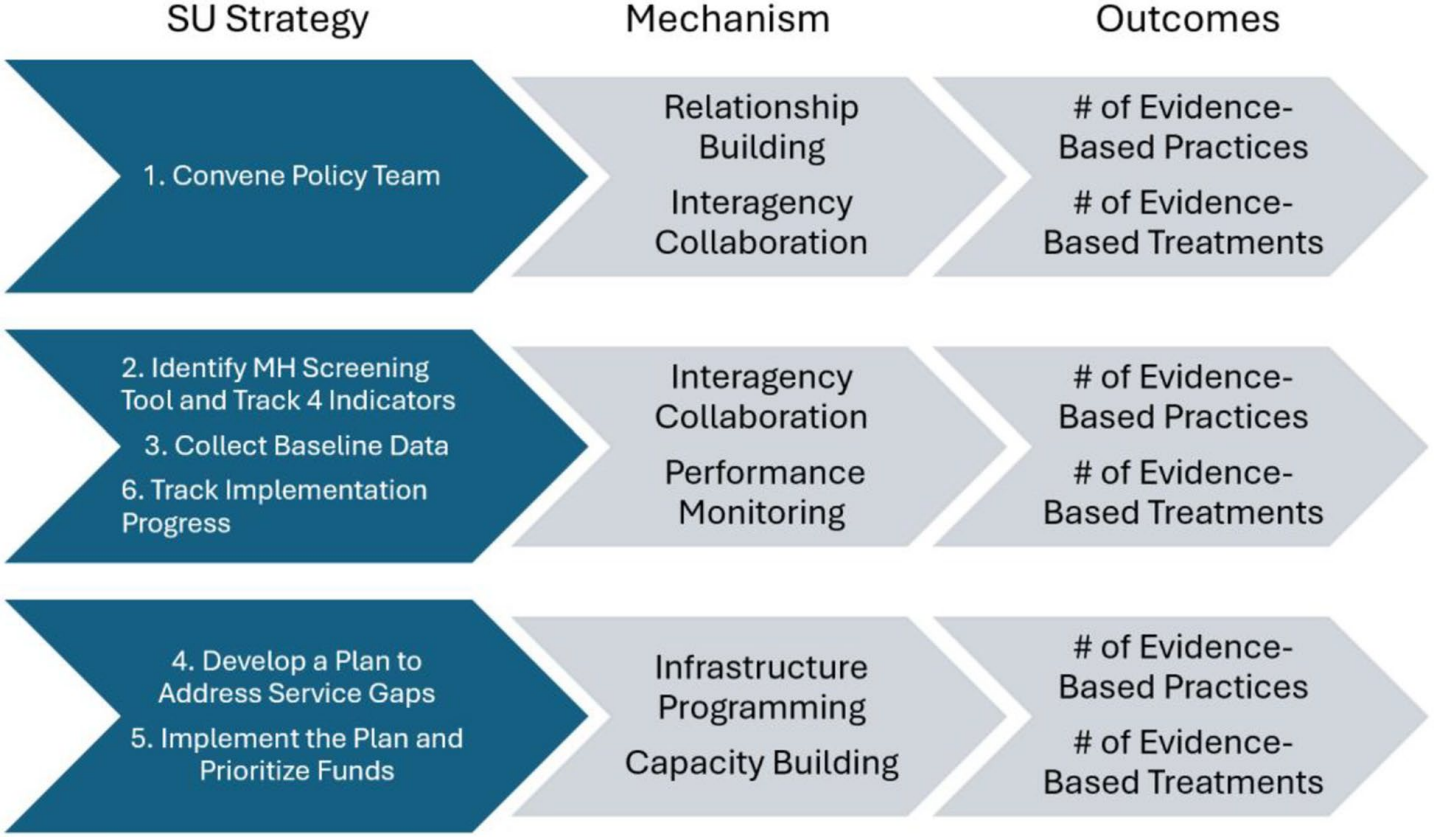
SU Strategies and Implementation Mechanisms

Implementation science increasingly prioritizes the study of implementation mechanisms to better understand how efforts to improve services function. The study of mechanisms examines how a strategy operates within a given context. As Geng and colleagues [7] note, studying strategies helps to 'determine what information from new contexts is needed to infer across contexts, and if that information is available, what those effects would be—thereby advancing generalization in implementation research' (pp. 1–2). Relatively little is known about implementation strategies at the intersection of community MH services and local jails and probation systems or the mechanisms to actualize these strategies. This manuscript presents a planned analysis of implementation strategies to assess the mechanisms influencing different outcomes and the environmental features that enable these mechanisms to have an impact—a key goal of the Implementation Mechanisms of Justice and Behavioral Health (IM Justice BH) study [3], as shown in Fig. 1. The mechanisms that are explored are relationship building among agencies on a policy team, performance monitoring (i.e., deciding on key agency metrics and tracking them over time to gauge service improvement efforts), and interagency coordination (such as regular meetings of county mental health, substance use, jail, and probation leaders, sharing resources, etc.) with counties using different strategies to implement these mechanisms. Other related mechanisms are capacity building by hiring staff to augment planning, programs, and analyses, and infrastructure programming to build quality services.

## Methods

This study investigates participation in Stepping Up, county characteristics, implementation mechanisms, and organizational factors that might explain the availability of criminal legal evidence-based practices (EBPs) and evidence-based mental health treatments (MH-EBTs) in a jurisdiction. The list of EBPs and MH-EBTs is derived from a consensus-building exercise conducted as part of this study [5] and is provided in Supplemental Table S1. Table S2 shows all the survey items used to define EBPs and MH-EBTs.

### Data Source

The main data for this research came from a survey of 519 U.S. counties conducted between 2020 and 2022 (contact the senior author for a copy of the instrument). This survey was the first wave of a 5-year study aimed at comparing implementation mechanisms and outcomes between counties that joined Stepping Up and matched pair counties that did not join this initiative [3, 8]. To identify matched control counties, we implemented a multilevel matching strategy that accounted for the hierarchical structure of counties nested within states. This involved estimating the likelihood of Stepping Up participation using logistic regression and machine learning models, and then applying a global optimization algorithm to select control counties with similar demographic, health, and justice-related characteristics. More details on the matching methodology can be found in Ramezani et al. [8]. A comprehensive analysis of baseline differences between Stepping Up and matched control counties — including health infrastructure, criminal legal indicators, and demographic characteristics — is available in Ramezani et al. [9]. The study contacted 950 counties (475 Stepping Up counties and 475 control counties), of which 519 completed the baseline survey questionnaire, yielding a 55% response rate at the county level. This is a relatively high response rate for organizational survey-related research [10, 11]. The response rate for Stepping Up counties was 69%, while 40% of the control counties responded to this survey.

Predictors used in this study are from a dataset previously compiled by this research team (see [3, 12] for a description of the survey and extant data sets).

### Assessments

#### Hypothesized implementation mechanisms

The implementation mechanisms examined in our study, shown in Table 1, consisted of five scales developed using factor analysis and each had a Cronbach alpha greater than 0.7 (Ramezani et al., paper in progress) including (1) relationship-building among agencies to coordinate policies and programs; (2) performance metrics monitoring to assess efforts to improve service delivery and decarceration; (3) interagency coordination of efforts to provide services and decarcerate; (4) capacity building by hiring staff to augment planning, programs, and analyses; and (5) infrastructure programming to build quality services through specialized units, decision matrices for program placement, programs for higher risk individuals, and targeting specific needs. Although the original design was to include four mechanisms, we are using the five that were derived from factor analyses of the original survey questions [3]. That is, factor analyses revealed another a cohesive set of mechanisms that counties used to advance their reforms. Table 1 provides a definition for each strategy and descriptive statistics. Table S3 shows the survey items used to measure and created these implementation strategies scales.

**Table 1:** Descriptive Statistics of Response and Implementation Mechanisms

**Dependent Variables**		**Total**		**SU**		**Non-SU**	
**Continuous and Count Response Variables**		**Mean**	**S. D**	**Mean**	**S. D**	**Mean**	**S.D**
Number of EBPs (Policies/Program)	Number of criminal legal evidence-based programs/policies available in a county	12.97	4.41	13.66	4.050	11.77	4.75
Number of MH-EBTs	Number of evidence-based MH treatments available	29.54	11.69	30.34	11.16	28.15	12.49
**Predictor Variables**		**Total**		**SU**		**Control**	
**Implementation Strategy Scales**		**Mean**	**SD**	**Mean**	**SD**	**Mean**	**SD**
Relationship Building Range = (1, 4)	*Policy/Team Relationship Building Scale:* Agencies build relationships through sharing information, collaborative problem-solving, using or providing technical assistance to improve cross-agency assessment and screening, staff training, etc	2.54	0.75	2.67	0.71	2.30	0.76
Performance Monitoring Measure Range = (0, 15)	Respondent indicates that their agency tracks measures for populations with mental illness: (1) number of people with mental illnesses who are booked into jail, (2) average length of stay in jail, (3) percent linked to community-based treatment after release from jail; and (4) return to jail rate (i.e., recidivism). and (5) length of stay post jail treatment	4.46	4.67	5.10	4.86	3.28	4.04
Interagency Coordination Range = (1, 18)	*Interagency Relationship Coordination Scale:* Level of collaboration including sharing information, developing eligibility criteria, pooling funding or staff, sharing oversight, implementing cross-training, and developing protocols	10.88	3.91	11.12	3.81	10.46	4.04
Capacity Building Range = (1, 4)	*Capacity Building Scale:* Improve service capacity of different agencies within counties by hiring experts, therapists, psychiatrists, peer support specialists, boundary spanner working in more than one agency, and/or county coordinator, and obtaining behavioral health licensing	2.06	0.78	2.16	0.77	1.88	0.75
Infrastructure Programming (Quality) Range = (1, 4)	*Infrastructure Programming Scale:* Agencies used strategies to expand services for justice-involved individuals with mental illness and improve mental health or substance use services for justice-involved individuals	2.16	0.63	2.34	0.66	1.90	0.67

#### Covariates

County-level socio-demographic and general service variables were used as covariates in all analyses. They were selected based on the literature and previous empirical studies of their relationship with the evidence-based programs and treatment-related outcome variables [9]. Backward variable selection method was used to narrow down the variables used in the final models. We checked for multicollinearity among variables using a correlation matrix and the variance inflation factor index. We excluded variables that were highly correlated with the rest of the variables [13] after ensuring that their variance is explained through other variables included in the models.

Table 2 summarizes the county characteristics used as covariates in our models. Ten county sociodemographic characteristics were used, including a categorical variable for county size (< 250,000, between 250,000 and 750,000, and over 750,000 county population, indicating county sizes small, medium, and large, respectively), whether the county was located in a rural geographic region, percentage of Hispanic and Black population in the county, per capita county jail population size, MH provider rate (psychiatrists, psychologists, counselors, and social workers), primary care physician rate, whether agencies in the county received Medicaid funding, and whether the county was a designated medically underserved county.

**Table 2:** Characteristics of the Counties

	**Total**		**SU**		**Control**	
**Continuous predictors**	**Mean**	**SD**	**Mean**	**SD**	**Mean**	**SD**
Jail Pop Per Capita^1^	0.00328	0.00199	0.00329	0.00201	0.00327	0.00195
%Hispanic & Black	19.42	18.23	21.19	19.19	16.38	16.04
Primary Care Physicians Rate^2^	68.23	30.20	70.87	30.61	63.61	28.99
MH Providers Rate^2^	186.35	135.94	202.71	148.03	157.99	106.50
Ratio of Agencies in the County Receiving Medicaid Funding	0.43	0.45	0.42	0.44	0.45	0.46
Total Number of Staff^3^	143	369	159	405	114	294
**Categorical predictors**	**Frequency**	**Proportion**	**Frequency**	**Proportion**	**Frequency**	**Proportion**
Rural County	Rural: 88 Urban: 431	0.170 0.830	40 288	0.122 0.878	48 143	0.251 0.749
Large County^4^	48	0.092	39	0.119	9	0.047
Medium County^4^	103	0.198	75	0.229	28	0.147
Small County^4^	368	0.709	214	0.652	154	0.806
Medically Underserved (MU)^5^	MU: 103 Not MU: 398	0.206 0.794	53 269	0.165 0.835	50 129	0.279 0.721
Stepping Up (SU)	SU: 328 Not SU: 191	0.632 0.368	-	-	-	-

^1^Jail population per capita calculated by dividing total jail population by county population. Values reported to five decimal places to reflect meaningful variation.

^2^Rates per 100,000 county residents.

^3^Includes staff involved in behavioral health and justice coordination. Reflects the number of staff hired by county agencies in the past year.

^4^County size defined by population: Small < 250,000; Medium 250,000–750,000; Large > 750,000.

^5^Defined using HRSA criteria for medically underserved areas (Health Resources & Services Administration, 2023).

#### Outcomes

Implementation outcomes in this study included: (1) number of evidence-based practices (EBP) and (2) number of evidence-based treatments (MH-EBT) for individuals with mental illness involved with justice systems. Definitions are provided in Table 1 with descriptive statistics.

#### Stepping Up status

Stepping Up status was derived from the Stepping Up website listing all counties that have signed a resolution to be part of the initiative at the time of data collection.

### Statistical analysis

Multilevel regression (i.e. hierarchical linear model [HLM]) and mediation analyses were employed to examine: (1) the impact of Stepping Up on various service delivery outcomes; (2) influence of implementation mechanisms on each outcome; and (3) the role of implementation mechanisms as mediators of the effects of Stepping up on implementation outcomes. As recommended by Raudenbush and Bryk [14], we calculated the intraclass correlation coefficient (ICC), reflecting counties nested within states, as an initial step to determine whether performing multilevel models was necessary. ICC values ranged around 0.1 and, as recommended by Lee [15] for an intraclass correlation of 0.1 and above, we used HLM nesting counties within states. Analyses employed a two-level generalized hierarchical linear model (GHLM) to account for the clustering of counties within states, addressing potential correlations among responses within states, and the hierarchical nature of our data. Because EBPs and MH-EBPs consisted of count measures, which followed a Poisson distribution, GHLM using the Poisson family and a logarithmic link function, adding the state clustering effect, were used [16]. To facilitate interpretation of effect estimates, we exponentiated model coefficients, presenting results as incident rate ratios (IRRs).” An exploratory mediation analysis was employed using the Sobel test [17] to examine potential indirect effects. R [18] was used to conduct all analyses. Lme4 [19] and bda [20] packages in R were used for the analyses. A significance level of 0.05 was used for the analysis.

## Results

The average number of EBPs was 13.0 out of 18 and the average number of MH-EBTs (treatments) was 29.6 out of 42 (see Table 1). The implementation strategies are also presented in Table 1. Nearly 70 percent of counties reported the use of *relationship building* mechanisms with other relevant agencies; this mechanism had a comparatively high mean response compared to other implementation mechanisms. Around 38 percent of the counties reported using *performance monitoring* mechanisms, 38 percent reported the use of *interagency coordination across agencies*, 43.5 percent reported using *capacity-building efforts*, and 51.4 percent reported the use of *infrastructure* programming mechanisms to provide quality services*.*

### Impact of stepping up on EBP and MH-EBTs

Stepping Up status significantly predicted both more EBPs and more MH-EBTs in models estimated using a log link function appropriate for count outcomes. These models controlled for rurality, county size, medically underserved area, percent of Hispanic and Black population, primary care physicians’ rate, jail population per capita, MH provider rate, and Medicaid funding for services in the counties (Table 3). Being a Stepping Up county is positively associated with EBPs (p = 0.002) and MH-EBTs (p = 0.018). These correspond to IRRs of 1.0946 (95% CI: [1.0336, 1.1591]) for EBPs and 1.0471 (95% CI: [1.0087, 1.0869]) for MH-EBTs, indicating approximately 9.5% and 4.7% higher rates in Stepping Up counties compared to non-participating counties, respectively. Detailed models with all the coefficients can be found in Supplemental Table S4.

**Table 3:** HLM models predicting EBPs and MH-EBTs based on SU Status

		Model 1: Criminal Legal EBPs (18 programs/practice) 435 counties in 40 states					Model 2: MH-EBTs (42 MH treatments) 435 counties in 40 states			
	Estimate	SE	IRR	95% CI (IRR)	P	Estimate	SE	IRR	95% CI (IRR)	P
SU Status	0.0904	0.0293	1.0946	[1.0336, 1.1591]	0.002**	0.046	0.0195	1.0471	[1.0087, 1.0869]	0.0185*
		These models controlled for Rurality, county Size, medically underserved area, Percent of Hispanic and Black population, primary care physicians’ rate, jail population per capita, MH provider rate, and Medicaid funding for services								

^*^ indicates a significance < 0.05 ** indicates a significance <.01 *** indicates a significance <.001

Note: Models were estimated using a log link function appropriate for count outcomes. Incidence Rate Ratios (IRRs) and 95% confidence intervals are exponentiated from log-scale coefficients to aid interpretability. Detailed models with all the coefficients can be found in Supplemental Table S4

### Impact of implementation mechanisms on EBP and MH-EBTs

Table 4 shows how implementation mechanisms predict implementation outcomes (EBPs and MH-EBTs) after controlling for rurality, county size, medically underserved area, percent of Hispanic and Black population, primary care physicians’ rate, jail population per capita, MH provider rate, and Medicaid funding for services in the counties. Performance monitoring (p = 0.0011) and interagency coordination (p < 0.001) were both significantly associated with greater implementation of EBPs and MH-EBTs. For EBPs, performance monitoring had an IRR of 1.0111 (95% CI: [1.0045, 1.0177]), indicating a 1.1% increase in this outcome. Interagency coordination had an IRR of 1.0185 (95% CI: [1.0098, 1.0273]), indicating a 1.85% increase in EBPs. For MH-EBTs, performance monitoring was a significant predictor (p < 0.001) with an IRR of 1.0113 (95% CI: [1.0068, 1.0159]), indicating a 1.13% increase, and interagency coordination was another significant predictor (p < 0.001) an IRR of 1.0159 (95% CI: [1.0099, 1.0219]), indicating a 1.59% increase. This suggests that both structured monitoring and collaborative efforts among agencies contribute to the broader adoption of evidence-based practices and treatments Relationship building was significantly associated with MH-EBTs only (p = 0.0002), with an IRR of 1.0837 (95% CI: [1.0386, 1.1304]), with greater relationship building being associated with an 8.37% increase in MH-EBTs. Neither infrastructure programming nor capacity building predicted number of EBPs or MH-EBTs in these covariate-adjusted models. Detailed models with all the coefficients can be found in Supplemental Table S5.

**Table 4:** HLM models predicting EBPs and MH-EBTs based on Implementation Mechanisms

		Model 1: Criminal Legal EBPs (18 programs/practice) 435 counties in 40 states					Model 2: MH-EBTs (42 MH treatments) 435 counties in 40 states			
	Estimate	SE	IRR	95% CI (IRR)	P	Estimate	SE	IRR	95% CI (IRR)	P
Relationship building	0.0454	0.0322	1.0465	[0.9829, 1.1142]	0.1591	0.0804	0.0219	1.0837	[1.0386, 1.1304]	0.0002***
Performance monitoring measure	0.011	0.0034	1.0111	[1.0045, 1.0177]	0.0011**	0.0112	0.0023	1.0113	[1.0068, 1.0159]	< 0.001***
Interagency coordination	0.0183	0.0044	1.0185	[1.0098, 1.0273]	< 0.001***	0.0158	0.003	1.0159	[1.0099, 1.0219]	< 0.001***
Infrastructure programming	0.0521	0.0358	1.0535	[0.9827, 1.1296]	0.1454	0.0222	0.0243	1.0225	[0.9749, 1.0723]	0.3615
Capacity building	0.0275	0.023	1.0279	[0.9836, 1.0749]	0.2334	0.0273	0.0158	1.0277	[0.9974, 1.0591]	0.0843
		These models controlled for rurality, county Size, medically underserved area, Percent of Hispanic and Black population, primary care physicians’ rate, jail population per capita, MH provider rate, and Medicaid funding for services								

^*^ indicates a significance < 0.05 ** indicates a significance <.01 *** indicates a significance <.001

Note: Models were estimated using a log link function appropriate for count outcomes. Incidence Rate Ratios (IRRs) and 95% confidence intervals are exponentiated from log-scale coefficients to aid interpretability. Detailed models with all the coefficients can be found in Supplemental Table S5

### Mediation analysis

#### Step 1: Exploratory analyses

When including both Stepping Up status and implementation mechanisms in the covariate-adjusted models, the direct effect of Stepping Up status on EBPs and MH-EBTs is no longer significant (see Table 5). The pattern of associations between relationship building implementation mechanisms and EPBs/MH-EBTs remained the same as when Stepping Up status was not included as a predictor in the models. Specifically, in predicting EBPs, performance monitoring (p = 0.001, IRR = 1.0110, 95% CI: [1.0044, 1.0177]) and interagency coordination (*p* < 0.001, IRR = 1.0186, 95% CI: [1.0099, 1.0274]) were both associated with greater EBPs, while Stepping Up status was no longer significant (p = 0.734, IRR = 1.0109, 95% CI: [0.9492, 1.0766]). In predicting MH-EBTs, relationship building strategies (*p* < 0.001, IRR = 1.0860, 95% CI: [1.0399, 1.1341]), performance monitoring (p < 0.001, IRR = 1.0116, 95% CI: [1.0070, 1.0162]) and interagency coordination (p < 0.001, IRR = 1.0155, 95% CI: [1.0094, 1.0216]) were all positively associated with MH-EBTs, while Stepping Up status is no longer significant (p = 0.125, IRR = 0.9676, 95% CI: [0.9270, 1.0099]). Infrastructure programming and capacity building remained non-significant predictors of EBPs and MH-EBTs. Detailed models with all the coefficients can be found in Supplemental Table S6.

**Table 5:** Full HLM models predicting EBPs and MH-EBTs based on SU Status and Implementation Mechanisms

		Model 1: Criminal Legal EBPs (18 programs/practice) 435 counties in 40 states					Model 2: MH-EBTs (42 MH treatments) 435 counties in 40 states			
	Estimate	SE	IRR	95% CI (IRR)	P	Estimate	SE	IRR	95% CI (IRR)	P
SU Status	0.0108	0.0317	1.0109	[0.9492, 1.0766]	0.7337	−0.03298	0.02152	0.9676	[0.9270, 1.0099]	0.12547
Relationship building	0.0447	0.0323	1.0457	[0.9817, 1.1139]	0.1662	0.08249	0.02192	1.0860	[1.0399, 1.1341]	< 0.001***
Performance monitoring measure	0.0109	0.0034	1.0110	[1.0044, 1.0177]	0.0012***	0.0115	0.00232	1.0116	[1.0070, 1.0162]	< 0.001***
Interagency coordination	0.0184	0.0044	1.0186	[1.0099, 1.0274]	< 0.001***	0.01536	0.00306	1.0155	[1.0094, 1.0216]	< 0.001***
Infrastructure programming	0.0506	0.036	1.0519	[0.9810, 1.1285]	0.1602	0.02705	0.02452	1.0274	[0.9793, 1.0785]	0.26992
Capacity building	0.0274	0.023	1.0278	[0.9836, 1.0747]	0.2347	0.02769	0.01585	1.0281	[0.9977, 1.0596]	0.08066
		These models controlled for Rurality, county Size, medically underserved area, Percent of Hispanic and Black population, primary care physicians’ rate, jail population per capita, MH provider rate, and Medicaid funding for services								

^*^ indicates a significance < 0.05 ** indicates a significance <.01 *** indicates a significance <.001

Note: Models were estimated using a log link function appropriate for count outcomes. Incidence Rate Ratios (IRRs) and 95% confidence intervals are exponentiated from log-scale coefficients to aid interpretability. Detailed models with all the coefficients can be found in Supplemental Table S6

These results suggest that Stepping Up status may have an indirect effect on the EBPs and MH-EBTs through some implementation mechanisms. Therefore, mediation analyses were performed to test this indirect effect.

#### Step 2: Mediation models

**Fig. 2:**
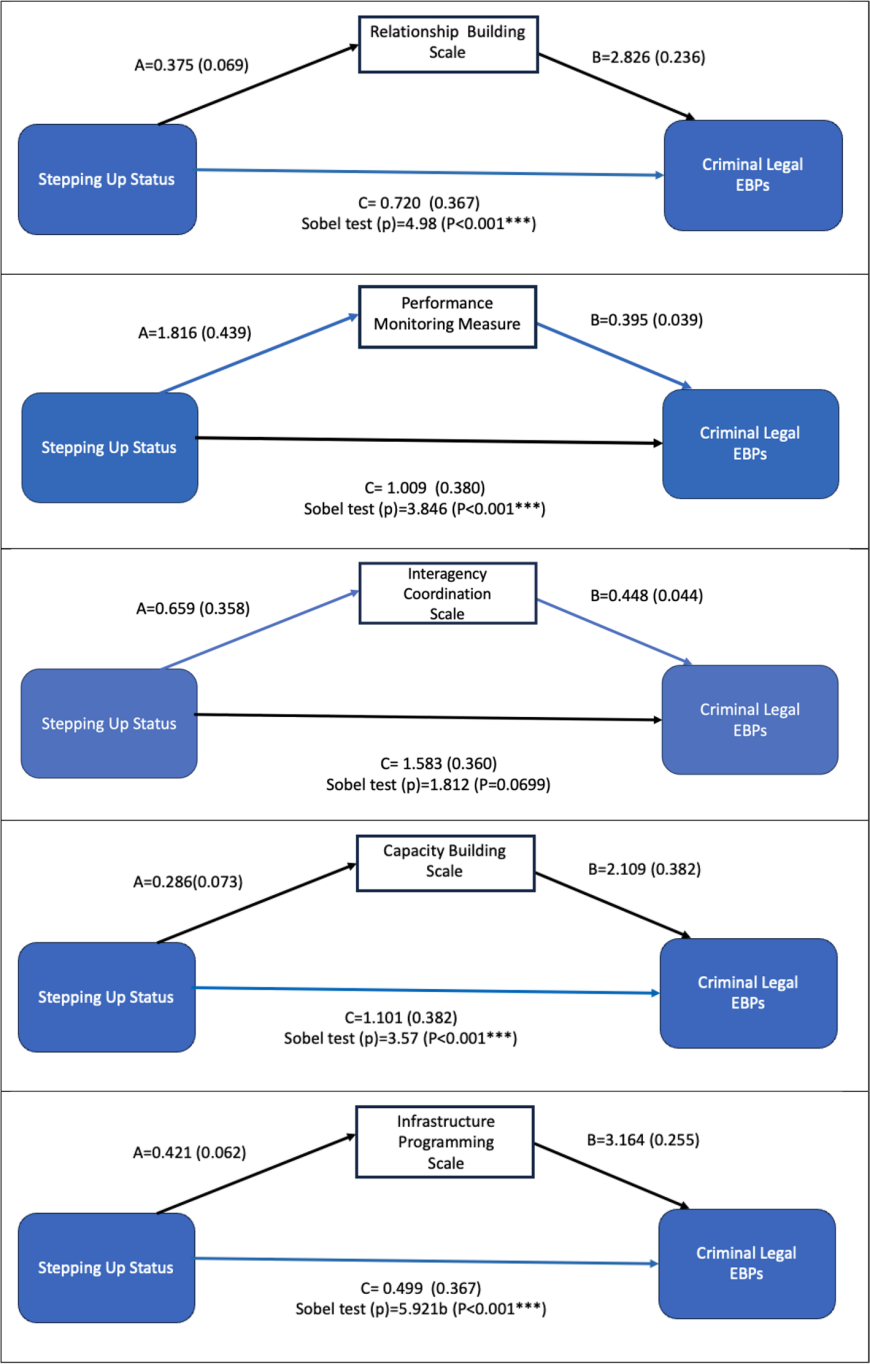
Mediation models predicting the number of criminal legal EBPs

When predicting the EBPs via each implementation mechanism (see Fig. 2 for relationship building, performance monitoring, interagency coordination, capacity building, and infrastructure programming mechanisms), Stepping Up status had a significant indirect/mediated effect on the criminal legal EBPs via four scales. The indirect effect between Stepping Up status and EBPs was significant via the relationship building scale (p < 0.001), performance monitoring scale (p < 0.001), infrastructure programming scale (*p* < 0.001), and capacity building scale (*p* < 0.001). This indirect effect was approaching statistical significance via the interagency coordination scale (p-value = 0.07).

**Fig. 3:**
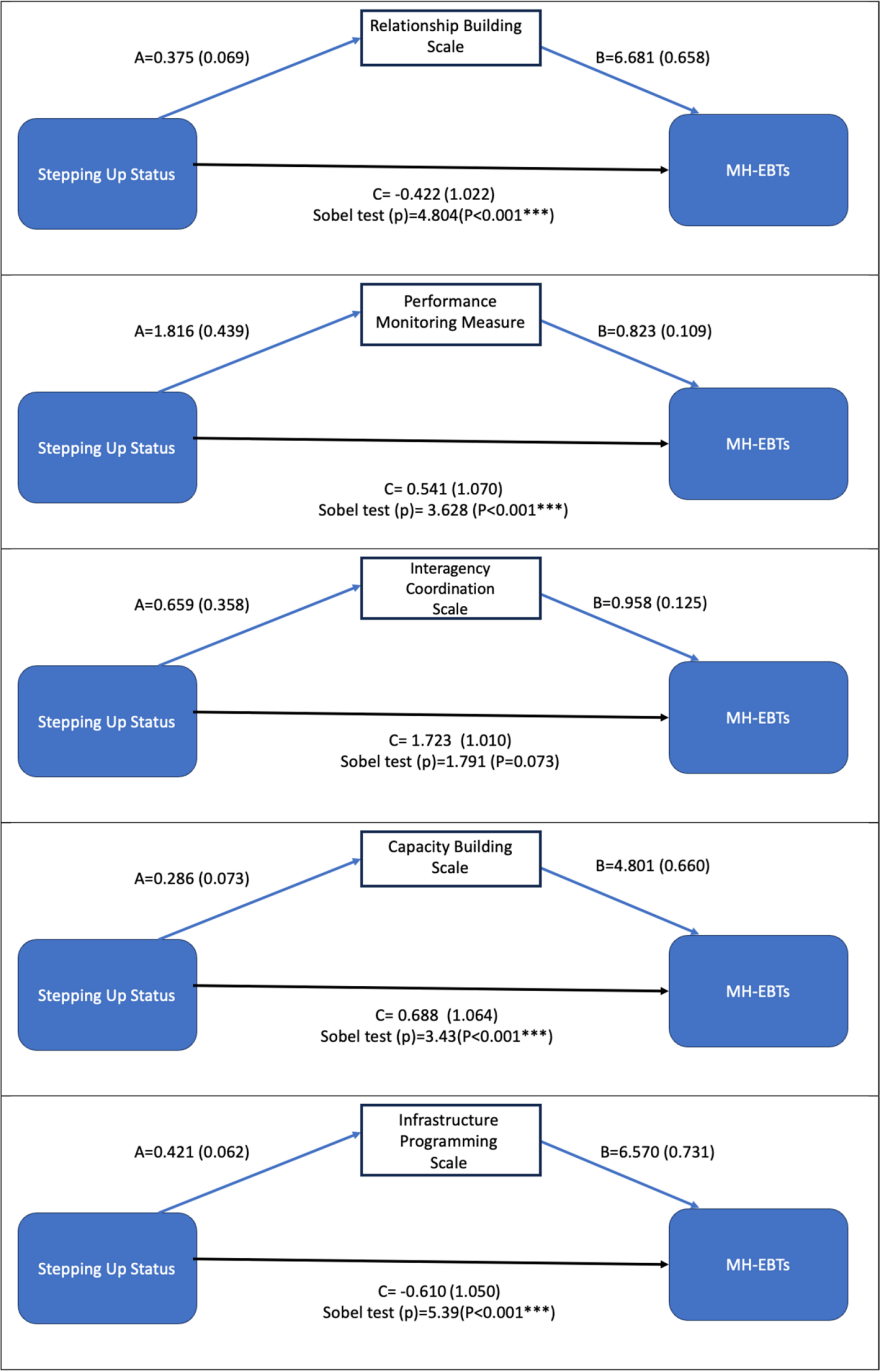
Mediation models predicting the number of MH-EBTs

When predicting the MH-EBTs via implementation mechanism (see Fig. 3), Stepping Up status had a significant indirect/mediated effect on the MH-EBTs like EBPs. The indirect effect between Stepping Up status and MH-EBTs was significantly mediated via the relationship building scale, performance monitoring measure (p < 0.001), capacity building scale (p-value < 0.001), and infrastructure programming implementation mechanisms (p-value < 0.001). This indirect effect was not statistically significant through the interagency coordination scale (*p*-value = 0.07).

### Demographic variables associated with implementation outcomes

The results of two HLM regression models, which show results for covariates in models predicting the number of EBPs and MH-EBTs, are presented in Supplementary Table S6. For EBPs, Medicaid funding for services was the only significant demographic predictor, with an IRR = 1.0970 (95% CI: [1.0275, 1.1717]), indicating a 9.7% increase. For MH-EBTs, significant demographic predictors included: county size (medium vs. large), with an IRR = 1.0959 (95% CI: [1.0210, 1.1767]), indicating a 9.6% increase; primary care physician rate, with an IRR = 1.0016 (95% CI: [1.0008, 1.0024]), indicating a 0.16% increase per unit; jail population per capita, with an IRR = 113,952 (95% CI: [93.3, 1.4e + 08]), indicating a very large effect likely due to scaling; mental health provider rate, with an IRR = 0.9997 (95% CI: [0.9995, 0.9999]), indicating a 0.03% decrease per unit; and Medicaid funding for services, with an IRR = 1.1496 (95% CI: [1.0959, 1.2060]), indicating a 14.96% increase.

## Discussion

Over 582 counties in 40 states have joined the Stepping Up Initiative, one of the largest reform efforts devoted to decarceration of individuals with MH disorders from jail and increasing the capacity in the community for providing MH services. This analysis demonstrated that, controlling for covariates, use of performance monitoring, interagency coordination and relationship building implementation mechanisms predicted having more MH-EBTs in counties, whereas performance monitoring and interagency coordination slightly predicted more EBPs. Furthermore, controlling for covariates, being in Stepping Up was associated with having more EBPs and mental health EBTs in a county. Finally, exploratory mediation analysis suggests that the effects of Stepping Up on the number of EBPs and mental health EBTs may be influenced by all mechanisms except for interagency coordination. SU is a robust predictor of both outcomes; more importantly the implementation mechanisms are more present and predictive in the presence of SU counties.

Intriguingly, relationship-building was not found to be a significant implementation mechanism for EBPs, contradicting some prior evidence [21]. This may be due to the inclusion of the interagency coordination and performance monitoring scales in the multivariate models, both which had statistically significant associations with EBPs and MH-EBTs and larger z scores than other mechanisms. Although their back-transformed effects had modest (1–2% increases), these mechanisms may reflect structured or operationalized forms of relationship building. Essentially, one can consider performance monitoring and interagency coordination to reflect or build upon healthy interagency relationships (relationship building), since both mechanisms require functional policy teams to make strides. Thus, in prior analyses, relationship-building may have served as a proxy for actual relationships [21]. From an implementation perspective, relationship building contributes to interagency policy teams making a commitment to reform and to make strides in difficult policy arenas [21, 22]—especially in addressing system issues that affect screening, identification, and placement of individuals with need in appropriate services. Policy teams need to have sufficient infrastructure to support their initiatives [22].

It is apparent that the way counties pursue policy and practice change uses key mechanisms of relationship-building, interagency coordination, and performance measures. The implication is that pursuing reforms can bridge inner and outer settings in terms of service delivery. Interagency coordination is needed to define target populations for decarceration, screening and assessment tools that are considered useful by multiple agencies and therefore used in decision-making, treatment placement criteria, and efforts to monitor treatment participation. Coordination can include networking, each agency acknowledging or accepting the revised process, or each agency aligning new practices to working towards common goals such as improving screening, referral, and treatment processes. Coordination is more than collaboration; in the former, the notion is that participating organizations are aligning their efforts. From a policy perspective, this means that the organizations agree to integrate some of their practices.

Performance monitoring was found to be a modest mechanism to advance the adoption of evidence-based practices and MH treatments. The SU initiative specified key metrics to guide counties in assessing their progress such as (1) the number of people with mental illnesses who are booked into jail, (2) average length of stay, (3) the percentage of people connected to community-based treatment after release from jail, and (4) rate of return to jail. These metrics assist in identifying the population of interest and defining key factors that affect the goal of decarceration. Even though the SU technical assistance providers prescribed what they considered to be useful metrics, developing and using these metrics appears to be difficult to achieve, given that slightly less than a third of the counties indicate progress on all four metrics. In a separate study, Hailemariam et al. [23] qualitatively examined use of performance metrics in 52 counties. The interviews revealed that there is a tremendous variation in what administrators/staff define as data. Most respondents assessed their use of data as rudimentary. While there was overall interest in using data to assess progress, most respondents did not have the capacity to track the four metrics and lacked the protocols to share data across agencies. Agencies were more likely to produce individual-level health or legal information, such as type of mental health illness, number of times entered the jail, instead of system-level measures like average length of stay or percentage of individuals with mental illness. The measures of length of stay or continuum of care are more difficult to calculate given the data systems’ limitations. For more counties to use performance metrics, attention should be given to updating management information systems, including developing electronic records that can be shared across agencies, standardized metrics, data dashboards, and training local staff to use the data.

This paper, along with Taxman et al. [24], reiterates the importance of interagency policy teams with good working relationships. Policy teams often exist but tend to focus on networking and information sharing and often do not have the tools to work across agency boundaries. The SU initiative tried to provide these tools through examples of memorandum of agreements that specify procedures for sharing information, for improving work performance, and means for dealing with conflict. The mechanisms were designed to extend relationship building to include collaboration or integrated service delivery. For example, some counties use existing policy groups such as Criminal Justice Coordination Commissions to improve policy and working relationships [25]. The Commissions usually have representatives from law enforcement, judicial, and correctional agencies as well as health and non-profit organizations. While the Commissions or similar groups can play a critical role, a survey of 489 individuals involved in Commissions acknowledged that the relationships exist, but the Commission is an underutilized mechanism to address system problems [25]. The SU initiative can be a conduit for using a Commission or for working on aligning policy across justice and health organizations. The mediation analyses illustrate that being part of Stepping Up reform can impact policies and practices in counties that have sufficient infrastructure to pursue the implementation mechanisms. This means that counties that pursue implementation mechanisms may be more likely to expand their use of EBPs and MH-EBTs than counties that are not in SU. Further analyses in this study will be done to better understand the role SU plays in reforms of policy and practice.

The value of the implementation mechanisms is supported by the findings that county characteristics such as county size, being part of the medically underserved counties, and other characteristics do not predict the EBPs and MH-EBTs. That is, how the agencies work together and what they do is more important than the size of the jurisdiction or the availability of sufficient reports. In SU, the technical assistance providers outlined the performance metrics that would be invaluable to assessing change in their system, including length of stay in jail, continued participation in community-based treatment, and recidivism. By providing this and offering guidance in terms of how to collect and report this data, the technical assistance (TA) providers guided counties; without such guidance the counties may have spent time trying to figure out what to measure. With SU, they knew what to measure and the TA assisted on data collection and analyses of these measures. TA providers would be advised to focus on performance metrics and interagency coordination as part of key reform effort. While efforts to educate stakeholders on the issues are useful, more emphasis should be placed on helping agencies coordinate and measure performance; both require the organizations and their actors to look externally instead of inward. And they require attention to being task-driven, where there is evidence that working together on an issue can produce observable outcomes [22, 26].

Study strengths include attention to the reform mechanisms that can impact EBPs and MH-EBTs outcomes which helps to identify forward processes that can affect building services in the community. Limitations of the study include its cross-sectional design, which aligns with our objective of examining associations rather than establishing causality. Our goal was not to make inferences regarding causal direction (i.e., whether increased use of implementation strategies leads to more EBPs/MH-EBTs or vice-versa). Given the study's design, determining reverse causation would require temporally ordered data, which cross-sectional methods do not allow for. As such, any speculation about causal pathways—whether direct or reverse—would exceed the scope of the data available. Future longitudinal research would be better suited to address such questions. Additionally, data collection occurred during COVID-19, when policy teamwork was more difficult due to lack of in-person events. It is possible that some counties were unable to work on decarceration efforts during this time. The survey was completed by a representative of the county, and it is possible that person did not know about the efforts other agencies were engaged with.

## Conclusion

Effective implementation mechanisms of performance measures and interagency coordination specifically can impact the number of EBPs and MH-EBTs available, particularly for counties that partake in Stepping Up. This study illustrates that enhanced treatment and practice reforms are feasible, but the internal mechanics of how reforms are pursued affects progress. And, counties that join SU, regardless of county size, are more likely to be effective in the implementation mechanisms to affect change. This suggests that technical assistance can facilitate better reform strategies, particularly when leadership supports reform efforts such as joining SU. Technical assistance can foster implementation strategies that can help counties overcome any limitations due to the nature and size of their county population, availability of funding, and availability of sufficient services and staffing. Efforts like Stepping Up strengthen reform efforts by providing a strategic path forward where the emphasis is on a data-driven reform framework. A data-driven strategy can be used to build or strengthen the agency’s working relationships, infrastructure, and data to review progress—all of which can lead to a commitment to quality services in and outside of the jail.

## Supplementary Information

Supplementary Material 1.

## Data Availability

The materials are available through the contact author, Dr. Faye S. Taxman.

## Abbreviations

SU: Stepping Up
EBPs: Evidence-Based Practices
MH-EBTs: Evidence-Based Mental Health Treatments
MH: Mental Health
HLM: Hierarchical Linear Model
ICC: Intraclass Correlation Coefficient
GHLM: Generalized Hierarchical Linear Model
TA: Technical Assistance
IRR: Incident Rate Ratios
