# Supplementary material for: Implementation mechanisms used in national efforts to improve community services to keep individuals with mental illness out of local jails
Source: Implement Sci Commun. 2025 Dec 13;7:15. doi: 10.1186/s43058-025-00835-5 (PMC12849348; doi:10.1186/s43058-025-00835-5)
Supplement: Supplementary file 1 — Supplementary Material 1. [file 43058_2025_835_MOESM1_ESM.docx]

**Supplemental Tables**

**Table S1:** *List of EBPs and MH-EBTs*

| \| **Criminal Legal EBPs (EBPs)** \| **Evidence-Based Treatments (MH-EBTs)** \| \| --- \| --- \| \| Diversion to mental health treatment \| Treatment for Cognitive - behavior therapy (CBT) for depression or Behavioral Activation for depression \| \| Problem solving courts \| Treatment for Interpersonal psychotherapy (IPT) for depression \| \| Therapeutic walk-in centers \| Treatment for selective serotonin reuptake inhibitors (SSRIs) for depression \| \| Crisis intervention \| Treatment for bipolar disorder or mania (including individual, group, medications, etc.) \| \| Crisis call-in centers \| Treatments for bipolar disorder or mania – Mood stabilizers for bipolar disorder or mania \| \| Mental health treatment required by court \| Treatments for bipolar disorder or mania – Education about bipolar disorder and its treatment (> 1 session) \| \| Building an alliance with patients \| Treatment for bipolar disorder or mania – Family education about bipolar disorder or family treatment for bipolar disorder \| \| Integrated mental health and substance use services \| Treatment for schizophrenia or psychosis (including individual, group, medications, etc.) \| \| Coordination between jail and community \| Treatment for schizophrenia or psychosis – CBT for psychosis \| \| Eligibility continuity \| Treatment for schizophrenia or psychosis – Family education about schizophrenia or psychosis \| \| Mental health peer navigators \| Treatment for schizophrenia or psychosis – Social skills training for schizophrenia \| \| Permanent supportive housing \| Treatment for schizophrenia or psychosis – First generation antipsychotics for psychosis \| \| Supported employment \| Treatment for schizophrenia or psychosis– First generation antipsychotics for psychosis \| \| Assertive Community Treatment (ACT), etc. \| Treatment for schizophrenia or psychosis – Second generation antipsychotics for psychosis \| \| Critical time Intervention \| Treatment for borderline personality disorder (including individual, group, medications, etc.) \| \| Family or caregiver education \| Treatment for borderline personality disorder – Dialectical behavior therapy (DBT) \| \| Trauma informed care \| Treatment and Psychopharmacology for borderline personality disorder \| \| Mental health training for correctional staff \| Treatment for post-traumatic stress disorder (PTSD); including individual, group, medications, etc. \| \|  \| Treatment for PTSD – CBT for PTSD or cognitive processing therapy for PTSD \| \|  \| Treatment for PTSD – Prolonged exposure for PTSD \| \|  \| Treatment for PTSD – Seeking Safety \| \|  \| Treatment for PTSD – SSRIs or tricyclic antidepressants for PTSD \| \|  \| Treatment for PTSD Anticonvulsants for re-experiencing \| \|  \| Treatment for anxiety (including individual, group, medications, etc.) \| \|  \| Treatment for anxiety – Exposure therapies or CBT for anxiety \| \|  \| Treatment for anxiety – SSRIs or tricyclic antidepressants for anxiety \| \|  \| Insomnia Group or individual counseling intervention (CBT, relaxation training, stimulus control training) \| \|  \| Group or individual counseling intervention for physical pain \| \|  \| Group or individual intervention addressing suicidal thoughts or behaviors \| \|  \| Interventions for suicidal thoughts or behaviors – DBT \| \|  \| Interventions for suicidal thoughts or behaviors – CBT for suicide prevention \| \|  \| Interventions for suicidal thoughts or behaviors – The Safety Planning Intervention \| \|  \| Interventions for substance use – Motivational interviewing for substance use \| \|  \| Interventions for substance use – Contingency management \| \|  \| Interventions for substance use – CBT for substance use \| \|  \| Interventions for substance use - Therapeutic community or residential treatment for substance use \| \|  \| Medications for opioid use disorders (e.g., Suboxone, Vivitrol) \| \|  \| Medications for opioid use disorders - Buprenorphine/Suboxone \| \|  \| Medications for opioid use disorders – Methadone \| \|  \| Medications for opioid use disorders - Naltrexone/Vivitrol \| \|  \| Medications for alcohol use disorders (e.g., Antabuse, Campral, Naltrexone) \| |
| --- | --- | --- | --- | --- | --- | --- | --- | --- | --- | --- | --- | --- | --- | --- | --- | --- | --- | --- | --- | --- | --- | --- | --- | --- | --- | --- | --- | --- | --- | --- | --- | --- | --- | --- | --- | --- | --- | --- | --- | --- | --- | --- | --- | --- | --- | --- | --- | --- | --- | --- | --- | --- | --- | --- | --- | --- | --- | --- | --- | --- | --- | --- | --- | --- | --- | --- | --- | --- | --- | --- | --- | --- | --- | --- | --- | --- | --- | --- | --- | --- | --- | --- | --- | --- |

**Table S2:** *Survey items used to define evidence-based practices List of Criminal Legal EBPs and MH-EBTs*

Question 16. Please identify whether each policy or practice listed below exists in your county and is available to serve justice-involved individuals.

Include any justice involved individuals identified through 911 calls, local law enforcement contact, pretrial jail detention, court appearances, specialty courts, jail sentences, probation, parole, community corrections, etc.

For a definition of the practice, please see the attached appendix.

| **Practices** | **Available to justice-involved individuals in your county?** |
| --- | --- |
| Diversion from any criminal justice action to mental health treatment make sure this is stated as police or court diversion | Yes, offered by my agency  Yes, offered by another agency  No  Not sure |
| Problem solving court (for example drug, mental health, veterans, or any other specialty court) | Yes, offered by my agency  Yes, offered by another agency  No  Not sure |
| Therapeutic walk-in or crisis centers or a place for police to drop off individuals in crisis (other than emergency rooms) | Yes, offered by my agency  Yes, offered by another agency  No  Not sure |
| Crisis Intervention Teams/police officers and social workers responding together (co-responding model), deflection programming | Yes, offered by my agency  Yes, offered by another agency  No  Not sure |
| Crisis call-in centers | Yes, offered by my agency  Yes, offered by another agency  No  Not sure |
| Mental health treatment required by the court | Yes, offered by my agency  Yes, offered by another agency  No  Not sure |
| Building an alliance with patients and taking patient preferences into account in mental health treatment planning | Yes, offered by my agency  Yes, offered by another agency  No  Not sure |
| Integrated mental health and substance use services; integrated dual disorder programs | Yes, offered by my agency  Yes, offered by another agency  No  Not sure |
| Coordination between jail and community mental health services at transitions into or out of jail (e.g., hand-off procedures) | Yes, offered by my agency  Yes, offered by another agency  No  Not sure |
| Eligibility continuity – a mechanism by which inmates can reactivate their Medicaid eligibility prior to release | Yes, offered by my agency  Yes, offered by another agency  No  Not sure |
| Mental health peer navigators, peer advocacy, or peer support | Yes, offered by my agency  Yes, offered by another agency  No  Not sure |
| Permanent supportive housing for individuals with mental health conditions | Yes, offered by my agency  Yes, offered by another agency  No  Not sure |
| Supported employment for individuals with mental health conditions | Yes, offered by my agency  Yes, offered by another agency  No  Not sure |
| Assertive community treatment (ACT), Forensic assertive community treatment (FACT), or Forensic Intensive Case Management (FICM) for mental illness | Yes, offered by my agency  Yes, offered by another agency  No  Not sure |
| Critical Time Intervention -OR- Case management for mental illness | Yes, offered by my agency  Yes, offered by another agency  No  Not sure |
| Family or caregiver education and support about the patient’s mental illness | Yes, offered by my agency  Yes, offered by another agency  No  Not sure |
| Trauma-informed care, settings, or services | Yes, offered by my agency  Yes, offered by another agency  No  Not sure |
| Mental health training for correctional staff | Yes, offered by my agency  Yes, offered by another agency  No  Not sure |

Question 17. Please identify whether each treatment listed below exists in your county and is available to serve justice-involved individuals.

Justice-involved can be identified through 911 calls, local law enforcement contact, pretrial jail detention, court appearances, specialty courts, jail sentences, probation, parole, and community corrections. For a definition of the program, please hover over the term. “Treatment” may include individual or group counseling, programs, and/or medications.

Most mental health treatments can be offered individually or in groups. Most are specific to the condition they are treating. For example, CBT for depression, CBT for psychosis, CBT for substance use, and CBT for criminal behavior or criminal thinking are all different treatments. If you are not sure if you offer CBT for a specific mental health disorder, please do not report it below.

For a definition of the practice, please see the attached appendix.

|  | **Available in your county?** |
| --- | --- |
| **Any treatment for depression (including individual, group, medications, etc.)** | Yes, offered by my agency  Yes, offered by another agency  No  Not sure |
| Cognitive-behavior therapy (CBT) for depression or Behavioral Activation for depression | Yes, offered by my agency  Yes, offered by another agency  No  Not sure |
| Interpersonal psychotherapy (IPT) for depression | Yes, offered by my agency  Yes, offered by another agency  No  Not sure |
| Selective serotonin reuptake inhibitors for depression | Yes, offered by my agency  Yes, offered by another agency  No  Not sure |
| **Any treatment for bipolar disorder or mania (including individual, group, medications, etc.)** | Yes, offered by my agency  Yes, offered by another agency  No  Not sure |
| Mood stabilizers for bipolar disorder or mania | Yes, offered by my agency  Yes, offered by another agency  No  Not sure |
| Education about bipolar disorder and its treatment (> 1 session) | Yes, offered by my agency  Yes, offered by another agency  No  Not sure |
| Family education about bipolar disorder or family treatment for bipolar disorder | Yes, offered by my agency  Yes, offered by another agency  No  Not sure |
| **Any treatment for schizophrenia or psychosis (including individual, group, medications, etc.)** | Yes, offered by my agency  Yes, offered by another agency  No  Not sure |
| Cognitive-behavioral therapy (CBT) for psychosis | Yes, offered by my agency  Yes, offered by another agency  No  Not sure |
| Family Education about schizophrenia or psychosis | Yes, offered by my agency  Yes, offered by another agency  No  Not sure |
| Social skills training for schizophrenia | Yes, offered by my agency  Yes, offered by another agency  No  Not sure |
| First generation antipsychotics (e.g., Phenothiazines (Chlorpromazine, Fluphenazine, Mesoridazine, Perphenazine, Thioridazine, Trifluoperazine), Haloperidol, Loxapine, Molindone, Thiothixene) for psychosis | Yes, offered by my agency  Yes, offered by another agency  No  Not sure |
| Second generation antipsychotics (e.g., Aripiprazole, Clozapine, Olanzapine, Quetiapine, Resperidone, Ziprasidone) for psychosis | Yes, offered by my agency  Yes, offered by another agency  No  Not sure |
| **Any treatment for borderline personality disorder (including individual, group, medications, etc.)** | Yes, offered by my agency  Yes, offered by another agency  No  Not sure |
| Dialectical behavior therapy (DBT) | Yes, offered by my agency  Yes, offered by another agency  No  Not sure |
| Psychopharmacology (SSRIs, mood stabilizers, low-dose antipsychotics) for borderline personality disorder | Yes, offered by my agency  Yes, offered by another agency  No  Not sure |
| **Any treatment for post-traumatic stress disorder (PTSD; including individual, group, medications, etc.)** | Yes, offered by my agency  Yes, offered by another agency  No  Not sure |
| Cognitive-behavioral therapy (CBT) for PTSD or cognitive processing therapy for PTSD | Yes, offered by my agency  Yes, offered by another agency  No  Not sure |
| Prolonged exposure for PTSD | Yes, offered by my agency  Yes, offered by another agency  No  Not sure |
| Seeking Safety | Yes, offered by my agency  Yes, offered by another agency  No  Not sure |
| SSRIs or tricyclic antidepressants for PTSD | Yes, offered by my agency  Yes, offered by another agency  No  Not sure |
| Anticonvulsants for re-experiencing | Yes, offered by my agency  Yes, offered by another agency  No  Not sure |
| **Any treatment for anxiety (including individual, group, medications, etc.)** | Yes, offered by my agency  Yes, offered by another agency  No  Not sure |
| Exposure therapies or cognitive-behavioral therapy (CBT) for anxiety | Yes, offered by my agency  Yes, offered by another agency  No  Not sure |
| SSRIs or tricyclic antidepressants for anxiety | Yes, offered by my agency  Yes, offered by another agency  No  Not sure |
| **Any group or individual counseling intervention for insomnia (CBT, relaxation training, stimulus control training)** | Yes, offered by my agency  Yes, offered by another agency  No  Not sure |
| **Any group or individual counseling intervention for physical pain** | Yes, offered by my agency  Yes, offered by another agency  No  Not sure |
| **Any group or individual intervention addressing suicidal thoughts or behaviors** | Yes, offered by my agency  Yes, offered by another agency  No  Not sure |
| Dialectical behavior therapy (DBT) for suicidal thoughts or behaviors | Yes, offered by my agency  Yes, offered by another agency  No  Not sure |
| Cognitive behavior therapy (CBT) for suicide prevention | Yes, offered by my agency  Yes, offered by another agency  No  Not sure |
| The Safety Planning Intervention | Yes, offered by my agency  Yes, offered by another agency  No  Not sure |
| **Any group or individual substance use intervention (not including medications, which we ask about later)** | Yes, offered by my agency  Yes, offered by another agency  No  Not sure |
| Motivational interviewing for substance use | Yes, offered by my agency  Yes, offered by another agency  No  Not sure |
| Contingency management | Yes, offered by my agency  Yes, offered by another agency  No  Not sure |
| Cognitive-behavioral therapy (CBT) for substance use | Yes, offered by my agency  Yes, offered by another agency  No  Not sure |
| Therapeutic community or residential treatment for substance use | Yes, offered by my agency  Yes, offered by another agency  No  Not sure |
| **Medications for opioid use disorders (e.g., Suboxone, Vivitrol)** | Yes, offered by my agency  Yes, offered by another agency  No  Not sure |
| Buprenorphine/Suboxone | Yes, offered by my agency  Yes, offered by another agency  No  Not sure |
| Methadone | Yes, offered by my agency  Yes, offered by another agency  No  Not sure |
| Naltrexone/Vivitrol | Yes, offered by my agency  Yes, offered by another agency  No  Not sure |
| **Medications for alcohol use disorders (e.g., Antabuse, Campral, Naltrexone)** | Yes, offered by my agency  Yes, offered by another agency  No  Not sure |

**Table S3.** *Survey Items Used to Measure Implementation Strategies*

Strategies Used By My County

**Question 26a.** **Does your county have an interagency workgroup that meets on a regular basis to work on issues related to reducing incarceration for individuals with mental illness? A regular basis refers to at a minimum of quarterly.**

**__Yes**

**__No**

**Question 26b. Check each box if you are aware that anyone in your county has used this strategy in the last 2 years to improve mental health or substance use services for justice-involved individuals and/or to reduce the number of people with mental illness in jail.**

|  | **Not working on this** | **Planning to address this** | **Some progress made** | **Significant progress made** |
| --- | --- | --- | --- | --- |
| 1) Worked with other agencies on county-wide planning to address the size of the jail population |  |  |  |  |
| 1. Implemented changes in a pilot before making changes system-wide. The pilot included trying out new approaches, measuring the impact, and then refining actual practice |  |  |  |  |
| 1. Hired experts: |  |  |  |  |
| 1. data experts |  |  |  |  |
| 1. county coordinator |  |  |  |  |
| 1. boundary spanner (works in more than one agency) |  |  |  |  |
| 1. psychiatrist(s) |  |  |  |  |
| 1. therapist(s) |  |  |  |  |
| 1. peer support specialist(s) |  |  |  |  |
| 1. other, indicate: |  |  |  |  |
| 1. Agreed on definitions of key terms guiding local efforts |  |  |  |  |
| - 1. We have a definition of mental illness and/or serious mental illness that is shared across agencies |  |  |  |  |
| - 1. We have a definition of substance use disorder or problematic substance use that is shared across agencies |  |  |  |  |
| - 1. We have a definition of recidivism that is shared across agencies |  |  |  |  |
| 1. Used strategies to expand services for justice-involved individuals with mental illness |  |  |  |  |
| - 1. Invested in pretrial programs for serious mental illness |  |  |  |  |
| - 1. Created specialized serious mental illness probation caseloads |  |  |  |  |
| - 1. Created decision matrices for processing serious mental illness cases |  |  |  |  |
| - 1. Focused investment on high risk, high need populations |  |  |  |  |
| - 1. Provided programming that targets criminogenic needs or dynamic risk factors related to an increased likelihood of recidivism |  |  |  |  |
| - 1. Eliminated criminal justice fees/fines |  |  |  |  |
| 6) Recruited and cultivated relationships with partners and other agencies to: |  |  |  |  |
| - 1. Promote information sharing |  |  |  |  |
| - 1. Engage in collaborative problem-solving |  |  |  |  |
| - 1. Create a shared vision/goal |  |  |  |  |
| 7) Developed, used, or updated a formal blueprint for improving mental health or substance use services for justice-involved individuals. |  |  |  |  |
| 8)Refined professional roles: |  |  |  |  |
| - 1. Identified champions for change in at least two agencies |  |  |  |  |
| - 1. Obtained Behavioral health licensing at state level |  |  |  |  |
| 9) Created linked data of clinical records from different agencies to facilitate improvement of mental health and substance use services across systems |  |  |  |  |
| 10) Provided or used local technical assistance to: |  |  |  |  |
| - 1. Develop consensus of goals and vision |  |  |  |  |
| - 1. Improve screening and assessment across agencies (e.g., share screening information across agencies) |  |  |  |  |
| - 1. Work on “hand-off” from one agency to another |  |  |  |  |
| - 1. Develop staff skills |  |  |  |  |
| - 1. Other, please specify: |  |  |  |  |
| 11) Captured and shared local knowledge through participation in activities to learn from other agencies or counties (e.g., examined lessons from other agencies, piloted a strategy used in another agency, conducted a site visit to another jurisdiction). |  |  |  |  |
| 12) Provided mental health training for: |  |  |  |  |
| 1. Correctional officers |  |  |  |  |
| 1. Probation/parole officers |  |  |  |  |
| 1. Police officers |  |  |  |  |
| 1. Court staff |  |  |  |  |
| 1. Interagency groups |  |  |  |  |

**Table S4.** *Detailed HLM models predicting EBPs and MH-EBTs based on SU Status*

|  |  | Model 1: Criminal Legal EBPs (18 programs/practice)  435 counties in 40 states | | | | |  | | Model 2: MH-EBTs  (42 MH treatments)  435 counties in 40 states | | | |
| --- | --- | --- | --- | --- | --- | --- | --- | --- | --- | --- | --- | --- |
|  | Estimate | | SE | IRR | 95% CI (IRR) | P | Estimate | SE | | IRR | 95% CI (IRR) | P |
| (Intercept) | 2.4687 | | 0.0783 | 11.800 | [10.107, 13.783] | <0.001*** | 3.2222 | 0.0587 | | 25.112 | [21.973, 28.697] | <0.001*** |
| SU Status | 0.0904 | | 0.0293 | 1.0946 | [1.0336, 1.1591] | 0.002** | 0.046 | 0.0195 | | 1.0471 | [1.0087, 1.0869] | 0.0185* |
| Rural | -0.0621 | | 0.0424 | 0.9398 | [0.8659, 1.0202] | 0.1426 | 0.0306 | 0.0277 | | 1.0311 | [0.9768, 1.0882] | 0.2701 |
| Size medium | 0.0367 | | 0.0494 | 1.0374 | [0.9409, 1.1431] | 0.4581 | 0.0874 | 0.0341 | | 1.0913 | [1.0206, 1.1679] | 0.0104* |
| Size small | -0.1454 | | 0.0529 | 0.8648 | [0.7756, 0.9641] | 0.006** | -0.107 | 0.0367 | | 0.8985 | [0.8356, 0.9663] | 0.0036** |
| Medically underserved area | -0.0728 | | 0.0396 | 0.9300 | [0.8593, 1.0072] | 0.066 | -0.0233 | 0.0266 | | 0.9770 | [0.9261, 1.0311] | 0.3809 |
| Percent of Hispanic and Black population | 0.0002 | | 0.001 | 1.0002 | [0.9982, 1.0022] | 0.8476 | 0.0004 | 0.0007 | | 1.0004 | [0.9990, 1.0018] | 0.6259 |
| Primary care physicians’ rate | 0.0008 | | 0.0005 | 1.0008 | [0.9998, 1.0018] | 0.1314 | 0.0013 | 0.0004 | | 1.0013 | [1.0005, 1.0021] | 0.0004*** |
| Jail population per capita^+^ | 5.8033 | | 8.156 | 330.0 | [0.0002, 6.6e+07] | 0.4768 | 12.4805 | 5.6288 | | 263,000 | [93.3, 741,000] | 0.0266* |
| MH provider rate | 0.0002 | | 0.0001 | 1.0002 | [0.9999, 1.0005] | 0.1573 | -0.0001 | 0.0001 | | 0.9999 | [0.9997, 1.0001] | 0.5712 |
| Medicaid funding for services | 0.0744 | | 0.0306 | 1.0773 | [1.0145, 1.1436] | 0.015* | 0.1219 | 0.0208 | | 1.1296 | [1.0841, 1.1772] | <0.001*** |
| State used as random (clustering) effect | Variance  0.0057 | | SD  0.0756 |  |  |  | Variance  0.0175 | SD  0.1321 | |  |  |  |

* indicates a significance <0.05 ** indicates a significance <.01 *** indicates a significance <.001

^+^ Jail population per capita is expressed as a small numeric rate, which results in a relatively large coefficient on the log scale. This is expected behavior in log-linked models, where variables with smaller scales yield larger coefficients to reflect proportional effects. The model appropriately accounts for these scaling differences, and the exponentiated IRRs remain interpretable as relative changes per unit increase in the original scale.

Note: Models were estimated using a log link function appropriate for count outcomes. Incidence Rate Ratios (IRRs) and 95% confidence intervals are exponentiated from log-scale coefficients to aid interpretability.

**Table S5.** *Detailed HLM models predicting EBPs and MH-EBTs based on Implementation Strategies*

|  |  | Model 1: Criminal Legal EBPs (18 programs/practice)  435 counties in 40 states | | | | |  | | Model 2: MH-EBTs  (42 MH treatments)  435 counties in 40 states | | | |
| --- | --- | --- | --- | --- | --- | --- | --- | --- | --- | --- | --- | --- |
|  | Estimate | | SE | IRR | 95% CI (IRR) | P | Estimate | SE | | IRR | 95% CI (IRR) | P |
| (Intercept) | 2.004 | | 0.1033 | 7.4226 | [6.0631, 9.0869] | <0.001*** | 2.7148 | 0.0736 | | 15.099 | [13.084, 17.421] | <0.001*** |
| Rural | -0.0049 | | 0.0442 | 0.9951 | [0.9110, 1.0869] | 0.9111 | 0.058 | 0.0296 | | 1.0597 | [1.0001, 1.1224] | 0.0504 |
| Size medium | -0.0027 | | 0.0516 | 0.9973 | [0.8981, 1.1071] | 0.9583 | 0.0917 | 0.0361 | | 1.0960 | [1.0211, 1.1769] | 0.011* |
| Size small | -0.0956 | | 0.0563 | 0.9088 | [0.8122, 1.0172] | 0.0893 | -0.0203 | 0.0393 | | 0.9799 | [0.9060, 1.0592] | 0.6061 |
| Medically underserved area | -0.0608 | | 0.0406 | 0.9410 | [0.8675, 1.0207] | 0.134 | -0.0032 | 0.0282 | | 0.9968 | [0.9423, 1.0536] | 0.9104 |
| Relationship building | 0.0454 | | 0.0322 | 1.0465 | [0.9829, 1.1142] | 0.1591 | 0.0804 | 0.0219 | | 1.0837 | [1.0386, 1.1304] | 0.0002*** |
| Performance monitoring measure | 0.011 | | 0.0034 | 1.0111 | [1.0045, 1.0177] | 0.0011** | 0.0112 | 0.0023 | | 1.0113 | [1.0068, 1.0159] | <0.001*** |
| Interagency coordination | 0.0183 | | 0.0044 | 1.0185 | [1.0098, 1.0273] | <0.001*** | 0.0158 | 0.003 | | 1.0159 | [1.0099, 1.0219] | <0.001*** |
| Infrastructure programming | 0.0521 | | 0.0358 | 1.0535 | [0.9827, 1.1296] | 0.1454 | 0.0222 | 0.0243 | | 1.0225 | [0.9749, 1.0723] | 0.3615 |
| Capacity building | 0.0275 | | 0.023 | 1.0279 | [0.9836, 1.0749] | 0.2334 | 0.0273 | 0.0158 | | 1.0277 | [0.9974, 1.0591] | 0.0843 |
| Percent of Hispanic and Black population | -0.0005 | | 0.0011 | 0.9995 | [0.9974, 1.0016] | 0.6607 | -0.0005 | 0.0008 | | 0.9995 | [0.9980, 1.0010] | 0.5055 |
| Primary care physicians’ rate | 0.0005 | | 0.0006 | 1.0005 | [0.9993, 1.0017] | 0.3433 | 0.0015 | 0.0004 | | 1.0015 | [1.0007, 1.0023] | 0.0001*** |
| Jail population per capita^+^ | 13.2785 | | 8.3631 | 5.89e+05 | [0.0002, 1.5e+12] | 0.1123 | 11.7673 | 5.8373 | | 1.29e+05 | [93.3, 1.8e+08] | 0.0438* |
| MH provider rate | 0.00002 | | 0.0001 | 1.0000 | [0.9998, 1.0002] | 0.8435 | -0.0003 | 0.0001 | | 0.9997 | [0.9995, 0.9999] | 0.0052** |
| Medicaid funding for services | 0.0926 | | 0.0332 | 1.0970 | [1.0275, 1.1717] | 0.0053** | 0.1396 | 0.0232 | | 1.1499 | [1.0963, 1.2061] | <0.001*** |
| State used as random (clustering) effect | Variance  0.0035 | | SD  0.0587 |  |  |  | Variance  0.0136 | SD  0.1165 | |  |  |  |

* indicates a significance <0.05 ** indicates a significance <.01 *** indicates a significance <.001

^+^ Jail population per capita is expressed as a small numeric rate, which results in a relatively large coefficient on the log scale. This is expected behavior in log-linked models, where variables with smaller scales yield larger coefficients to reflect proportional effects. The model appropriately accounts for these scaling differences, and the exponentiated IRRs remain interpretable as relative changes per unit increase in the original scale.

Note: Models were estimated using a log link function appropriate for count outcomes. Incidence Rate Ratios (IRRs) and 95% confidence intervals are exponentiated from log-scale coefficients to aid interpretability.

**Table S6.** *Detailed full* *HLM models predicting EBPs and MH-EBTs based on SU Status and Implementation Strategies*

|  |  | | Model 1: Criminal Legal EBPs  (18 programs/practice)  435 counties in 40 states | | | |  | | Model 2: MH-EBTs  (42 MH treatments)  435 counties in 40 states | | | |
| --- | --- | --- | --- | --- | --- | --- | --- | --- | --- | --- | --- | --- |
|  | Estimate | SE | | IRR | 95% CI (IRR) | P | Estimate | SE | | IRR | 95% CI (IRR) | P |
| (Intercept) | 2.0027 | 0.1034 | | 7.4176 | [6.0579, 9.0783] | <0.001*** | 2.71838 | 0.07365 | | 15.165 | [13.158, 17.478] | <0.001*** |
| SU Status | 0.0108 | 0.0317 | | 1.0109 | [0.9492, 1.0766] | 0.7337 | -0.03298 | 0.02152 | | 0.9676 | [0.9269, 1.0099] | 0.12547 |
| Rural | -0.0041 | 0.0442 | | 0.9959 | [0.9116, 1.0881] | 0.9266 | 0.05549 | 0.02967 | | 1.0571 | [0.9971, 1.1202] | 0.06143 |
| Size medium | -0.0027 | 0.0516 | | 0.9973 | [0.8979, 1.1073] | 0.9588 | 0.09163 | 0.03609 | | 1.0959 | [1.0210, 1.1767] | 0.01112* |
| Size small | -0.0962 | 0.0563 | | 0.9083 | [0.8098, 1.0192] | 0.0874 | -0.01922 | 0.03939 | | 0.9810 | [0.9053, 1.0627] | 0.6255 |
| Medically underserved area | -0.0603 | 0.0406 | | 0.9415 | [0.8691, 1.0193] | 0.1374 | -0.00479 | 0.0282 | | 0.9952 | [0.9403, 1.0527] | 0.8652 |
| Relationship building | 0.0447 | 0.0323 | | 1.0457 | [0.9817, 1.1139] | 0.1662 | 0.08249 | 0.02192 | | 1.0860 | [1.0399, 1.1341] | 0.00017*** |
| Performance monitoring measure | 0.0109 | 0.0034 | | 1.0110 | [1.0044, 1.0177] | 0.0012*** | 0.0115 | 0.00232 | | 1.0116 | [1.0070, 1.0162] | <0.001*** |
| Interagency coordination | 0.0184 | 0.0044 | | 1.0186 | [1.0099, 1.0274] | <0.001*** | 0.01536 | 0.00306 | | 1.0155 | [1.0094, 1.0216] | <0.001*** |
| Infrastructure programming | 0.0506 | 0.036 | | 1.0519 | [0.9810, 1.1285] | 0.1602 | 0.02705 | 0.02452 | | 1.0274 | [0.9793, 1.0785] | 0.26992 |
| Capacity building | 0.0274 | 0.023 | | 1.0278 | [0.9836, 1.0747] | 0.2347 | 0.02769 | 0.01585 | | 1.0281 | [0.9977, 1.0596] | 0.08066 |
| Percent of Hispanic and Black population | -0.0005 | 0.0011 | | 0.9995 | [0.9974, 1.0016] | 0.6425 | -0.00042 | 0.00077 | | 0.9996 | [0.9981, 1.0011] | 0.57967 |
| Primary care physicians’ rate | 0.0005 | 0.0006 | | 1.0005 | [0.9993, 1.0017] | 0.3491 | 0.00155 | 0.00039 | | 1.0016 | [1.0008, 1.0024] | <0.001*** |
| Jail population per capita^+^ | 13.3543 | 8.3207 | | 6.3e+05 | [0.0002, 1.6e+12] | 0.1085 | 11.63952 | 5.87427 | | 1.13e+05 | [93.3, 1.4e+08] | 0.04754* |
| MH provider rate | 0.00002 | 0.0001 | | 1.0000 | [0.9998, 1.0002] | 0.8638 | -0.00026 | 0.0001 | | 0.9997 | [0.9995, 0.9999] | 0.00769** |
| Medicaid funding for services | 0.0926 | 0.0332 | | 1.0970 | [1.0275, 1.1717] | 0.0053** | 0.1395 | 0.02325 | | 1.1496 | [1.0959, 1.2060] | <0.001*** |
| State used as random (clustering) effect | Variance  0.0034 | SD  0.0584 | |  |  |  | Variance  0.0137 | SD  0.1169 | |  |  |  |

* indicates a significance <0.05 ** indicates a significance <.01 *** indicates a significance <.001

^+^ Jail population per capita is expressed as a small numeric rate, which results in a relatively large coefficient on the log scale. This is expected behavior in log-linked models, where variables with smaller scales yield larger coefficients to reflect proportional effects. The model appropriately accounts for these scaling differences, and the exponentiated IRRs remain interpretable as relative changes per unit increase in the original scale.

Note: Models were estimated using a log link function appropriate for count outcomes. Incidence Rate Ratios (IRRs) and 95% confidence intervals are exponentiated from log-scale coefficients to aid interpretability.
